# Visuo-Vestibular Information Processing by Unipolar Brush Cells in the Rabbit Flocculus

**DOI:** 10.1007/s12311-015-0710-8

**Published:** 2015-08-18

**Authors:** Robert A. Hensbroek, Tom J. H. Ruigrok, Boeke J. van Beugen, Jun Maruta, John I. Simpson

**Affiliations:** Department of Neuroscience & Physiology, New York University Medical School, New York, NY 10016 USA; Department of Neuroscience, Erasmus MC Rotterdam, 3000 CA Rotterdam, Netherlands; ENT Department, St. Mary’s Hospital, London, W2 1NY UK; Brain Trauma Foundation, 1 Broadway, New York, NY 10004 USA

**Keywords:** Cerebellum, UBC, Flocculus, Velocity, Eye position, Acceleration

## Abstract

The unipolar brush cell (UBC) is a glutamatergic granular layer interneuron that is predominantly located in the vestibulocerebellum and parts of the vermis. In rat and rabbit, we previously found using juxtacellular labeling combined with spontaneous activity recording that cells with highly regular spontaneous activity belong to the UBC category. Making use of this signature, we recorded from floccular UBCs in both anesthetized and awake rabbits while delivering visuo-vestibular stimulation by using sigmoidal rotation of the whole animal. In the anesthetized rabbit, the activity of the presumed UBC units displayed a wide variety of modulation profiles that could be related to aspects of head velocity or acceleration. These modulation profiles could also be found in the awake rabbit where, in addition, they could also carry an eye position signal. Furthermore, units in the awake rabbit could demonstrate rather long response latencies of up to 0.5 s. We suggest that the UBCs recorded in this study mostly belong to the type I UBC category (calretinin-positive) and that they can play diverse roles in floccular visuo-vestibular information processing, such as transformation of velocity-related signals to acceleration-related signals.

## Introduction

With over 30 publications, Enrico Mugnaini and his colleagues not only laid the foundation but also designed the architecture of our knowledge about a most unusual type of cell that predominantly resides in the granular layer of the vestibulocerebellum and parts of the vermis [1–3]. The unipolar brush cell (UBC) is a relatively small cell type with a single brush-like dendrite. It receives an excitatory synaptic input in the form of either a single extrinsic mossy fiber rosette or an intrinsic mossy-fiber-like rosette from another UBC [4–6]. Mugnaini’s laboratory furthermore firmly established that UBCs come in two histochemical subtypes [7–10]. Type I is calretinin-positive and strongly expresses the beta-1 isoform of phospholipase C whereas type II is positive for the group I metabotropic glutamate receptor mGluR1α and expresses the beta-4 isoform of phospholipase C [7]. Type I and type II cells have somewhat different soma sizes and, in vitro, display different physiological characteristics and firing frequencies [8]. Despite the wealth of information about development, distribution, typology, and basic physiological characteristics of UBCs, it remains unclear how they contribute to cerebellar information processing or why they are especially abundant in the vestibulocerebellum and parts of the vermis. A recent in vitro study has proposed that the synaptic integration capabilities of UBCs may be specifically suited to contribute to the control of slow eye and head movements [11].

As yet, however, surprisingly little is known of the behavior of UBCs in behaving animals, and it is clear that knowledge obtained with in vitro recordings needs to be evaluated in the intact brain. As a start, we have demonstrated in both anesthetized rats and rabbits that cells with a characteristic spontaneous firing frequency with very low variability could consistently be morphologically identified as a UBC [12, 13]. Here, using this fingerprint, we examined in the flocculus of anesthetized and awake rabbits the modulation of such cells in response to visuo-vestibular stimulation. Some examples are provided that indicate that these presumed UBCs have a rather wide range of response types and, therefore, they may be involved in highly diverse modes of information processing.

## Methods

Dutch belted rabbits were used either in acute experiments under anesthesia or in chronic experiments while awake. The experiments were conducted in New York, conformed to the Principles of Laboratory Animal Care, and were approved by the Institutional Animal Care and Use Committee of the New York University School of Medicine. Acute experiments in 15 rabbits were performed as described earlier [12, 14]. Briefly, animals were anesthetized with an intramuscular injection of a mixture of ketamine (45 mg/kg) and xylazine (5 mg/kg) and mounted in a stereotactic frame with the nasal bone at 57° to the horizontal. The animal was placed on a heating pad to maintain a physiological body temperature. Anesthetic levels were monitored by the absence of palpebral reflexes and paw pinch withdrawal; supplemental doses were given as required. The dorsolateral cerebellum was exposed, and the flocculus was accessed with a glass microelectrode (tip diameter of 0.7–2 μm) oriented at 37°–27° to the vertical axis in a parasagittal plane. Signals were amplified, bandpass-filtered at 10 Hz/100 Hz to 10 kHz, captured, and stored for off-line analysis using a CED1401 data acquisition device and Spike2 software (Cambridge Electronics Design). Off-line analysis was performed with Spike2 (Cambridge Electronics Design) and Microsoft Excel software.

Two Dutch belted rabbits were prepared for chronic awake recording using sterile surgical techniques [14]. In short, animals were anesthetized with a mixture of acepromazine (0.1 mg/kg intramuscularly (i.m.)), ketamine (45 mg/kg i.m.), and xylazine (5 mg/kg i.m.) and received supplements as required. A pedestal was fixed to the skull. A craniotomy was performed over the left paramedian lobule of the cerebellum, and a metal recording chamber was fixed around the craniotomy by attaching it to the pedestal. This cylindrical chamber was oriented such that the entire extent of the left flocculus could be reached [14, 15]. The brain was covered by a silastic sheet, and the chamber was closed by a screw top. A search coil was implanted on the left eye to measure eye position. The animal was allowed a recovery period of at least 1 week during which it was habituated to the recording setup. Neural recordings were made with the same techniques and equipment as described above. When spontaneous activity was recorded, the eyes were stationary and centered in the orbit. Recording sessions generally ran for 4 h but were terminated if the animal showed signs of agitation. Between recording sessions, the brain was covered by a silastic sheet and the chamber was sealed.

Our previous studies in anesthetized rats and rabbits showed that units displaying a characteristic regular spontaneous firing frequency were morphologically identified as UBCs [12, 13]. Based on these studies, which involved various types of interneurons and later also Purkinje cells, a decision algorithm was constructed that accurately identified the majority of recorded units [12, 15]. In order to use the decision algorithm, a minimum of 60 s of spontaneous activity is typically desired to ensure that the statistical measures of the spontaneous spike pattern are stable. The decision algorithm classifies a cell as a particular cell type or as a “border cell.” Border cells are cells with firing statistics that are considered too similar to the firing characteristics of a particular cell type to be accurately classified. Once a cell is classified as a border cell, it is excluded from further analysis. For a positive identification as a UBC in both the anesthetized and the awake rabbit, the statistical measures must pass through the following steps in sequence [15]:

The first step in the decision algorithm classifies cells as granule cells. To be not considered a granule cell or a border cell at that point, the average firing frequency has to be larger than 0.6 Hz and the CVlog smaller than 0.34.

The second step classifies cells as Purkinje cells. Recordings accompanied by a complex spike and a pause >9 ms in simple spike firing after the complex spike are considered to be from a Purkinje cell and need no additional classification. In those instances where no complex spike is recorded [15], to be not considered a Purkinje cell or a border cell, the CV2 has to be smaller than 0.15 or the MAD has to be larger than 0.01.

The third step classifies cells as UBCs. To be considered a UBC, the CV2 has to be smaller than 0.24.

**Fig. 1:**
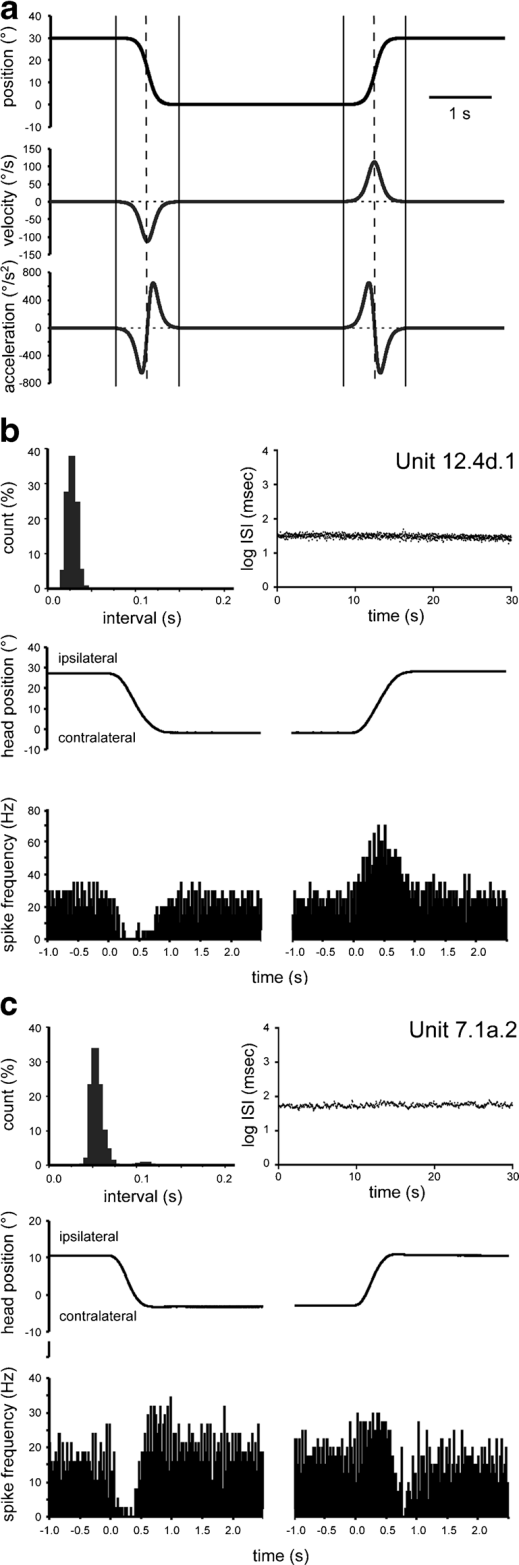
Response properties of floccular UBCs in the anesthetized rabbit. **a** Response kinematics of sigmoidal rotation. *Top trace*, head position; *middle trace*, head velocity, monophasic for each direction of rotation; *bottom trace*, head acceleration, biphasic for each direction of rotation. The *dashed vertical lines* indicate the peak velocity for each rotation direction. The *solid vertical lines* indicate the start and stop of the sigmoidal movement. **b** Example (unit 12.4d.1) demonstrating the narrow interspike interval (ISI) distribution. *Top left panel*, ISI histogram showing spike intervals during spontaneous activity expressed as normalized counts; *top right panel*, sequential log interspike intervals over a period of 30 s of spontaneous activity; *middle panel*, head position during sigmoidal stimulation, which was provided in the light by movement of the turntable by hand. The table movement provided by hand had a mean peak velocity and standard deviation of 64.5 ± 3.6° per second for contralateral movement and 67.9 ± 6.7° per second for ipsilateral movement; *bottom panel*, modulation profile (average of 7 cycles) in response to the corresponding head movement shown in the middle panel. The 0 reference in the time line indicates the onset of the turntable movement. Note the similarity of the modulation profile to the velocity trace in Fig. 1a. **c** Example (unit 7.1a.2) with a lower, but also regular firing pattern. Panels are arranged as in (**b**). The response to head movement was averaged over 16 cycles. The table movement provided by hand had a mean peak velocity and standard deviation of 42.8 ± 3.8° per second for contralateral movement and 41.0 ± 6.0° per second for ipsilateral movement. Note that the resulting modulation profile resembles the head acceleration profile, although shifted and spread out in time

Units identified as UBCs were recorded during visual-vestibular stimulation in the light and dark using sigmoidal rotation provided by a turntable. Sigmoidal stimulation mimics natural head movement [16] with a monophasic velocity profile and a biphasic acceleration profile (Fig. 1a). Compared to sinusoidal stimulation, sigmoidal stimulation allows for a clearer separation among position-, velocity-, and acceleration-related responses and thus provides improved detection of response asymmetries and comparison to the level of spontaneous activity.

## Results

Here we show some examples of UBC responses to sigmoidal visuo-vestibular stimulation of units that were recorded in the flocculus of either the anesthetized acute or the awake chronic rabbit. These units displayed regular spontaneous activity (without any applied visuo-vestibular stimulation) that adhered to the distinguishing characteristics of UBC identification demonstrated in ketamine/xylazine-anesthetized rats and rabbits [12, 15].

### Anesthetized Rabbit

In the ketamine/xylazine-anesthetized acute preparations, spontaneous-activity-identified UBCs generally had clear, but rather diverse patterns of modulation in response to sigmoidal stimulation. In general, UBCs were modulated with a 50–100-ms response delay and demonstrated little sensitivity to vision such that responses in the dark were almost always similar to responses in the light. Eye movements were not noted. Two main response types were recognized in relation to specific kinematics of the sigmoidal stimulation. The first type was related to the velocity profile. Figure 1b shows an example of such a cell (unit 12.4d.1). The characterizing measures for this cell and the others illustrated in this paper are listed in Table 1. The rather narrow ISI histogram of this cell is representative of a main characteristic of the UBCs in this study. This cell showed a decrease of activity when the head was rotated to the contralateral side and an increase of activity when the head was rotated to the ipsilateral side (a type 1 vestibular response). The activity returned to its resting level when the head was stationary in any turntable position. This particular cell had a symmetrical response pattern (about equal excitation and inhibition for oppositely directed rotations), but there were other units that showed only unidirectional responses (either excitation or inhibition for only one rotation direction). Also, some UBCs had a type 2 vestibular response (i.e., an increase of activity when moving to the contralateral side). Note that in our preparation, vertical semicircular canals were oriented so that they were minimally stimulated by rotation of the turntable.

**Table 1:** Relevant measures of spontaneous activity characteristics used to decide upon the UBC nature of the units shown in Figs. 1 and 2

Unit	Avg. firing (Hz)	CVlog	CV2	MAD
12.4d.1	33.7	0.040	0.069	0.0026
07.1a.2	18.2	0.028	0.149	0.0040
RB1.31b.2	17.8	0.035	0.063	0.0037
RB1.32.4	36.9	0.052	0.112	0.0016
RB1.11.6	26.1	0.037	0.125	0.0030

Measures for the decision algorithm were [1] the average firing frequency, [2] the CVlog (the coefficient of variation of the distribution of the natural logarithm of ISIs in milliseconds), [3] the CV2 (the mean of two times the absolute difference of successive ISIs divided by the sum of the two intervals), and [4] the median absolute difference (MAD) from the median ISI [15]

The second main response type was related to the acceleration profile of the sigmoidal stimulus. Figure 1c shows unit 7.1a.2 as an example. Here, the activity of the unit sharply decreased upon acceleration of the head in the contralateral direction. However, upon deceleration of that movement, the cell sharply increased its activity beyond the resting level. When the head was stationary, the activity stabilized. Accelerating the head in the ipsilateral direction increased activity whereas deceleration in this direction reduced activity. Note that the activity profile of this unit resembles the acceleration profile of the sigmoidal movement, as shown in Fig. 1a. Other units exhibited an acceleration profile for one movement direction, but a velocity profile for the opposite movement direction.

Of the 14 UBCs obtained from anesthetized rabbits, 11 UBCs modulated only to head velocity (6 type 1, 4 type 2, 1 type 3), 1 UBC modulated purely to head acceleration, and 2 UBCs modulated to a combination of head acceleration and head velocity (acceleration-related response to one rotation direction and velocity-related response to the other). For these 14 cells, the average firing rate was 18.9 ± 8.3 Hz (mean ± s.d.). The CV2 was 0.14 ± 0.12 (mean ± s.d.).

### Awake Rabbit

**Fig. 2:**
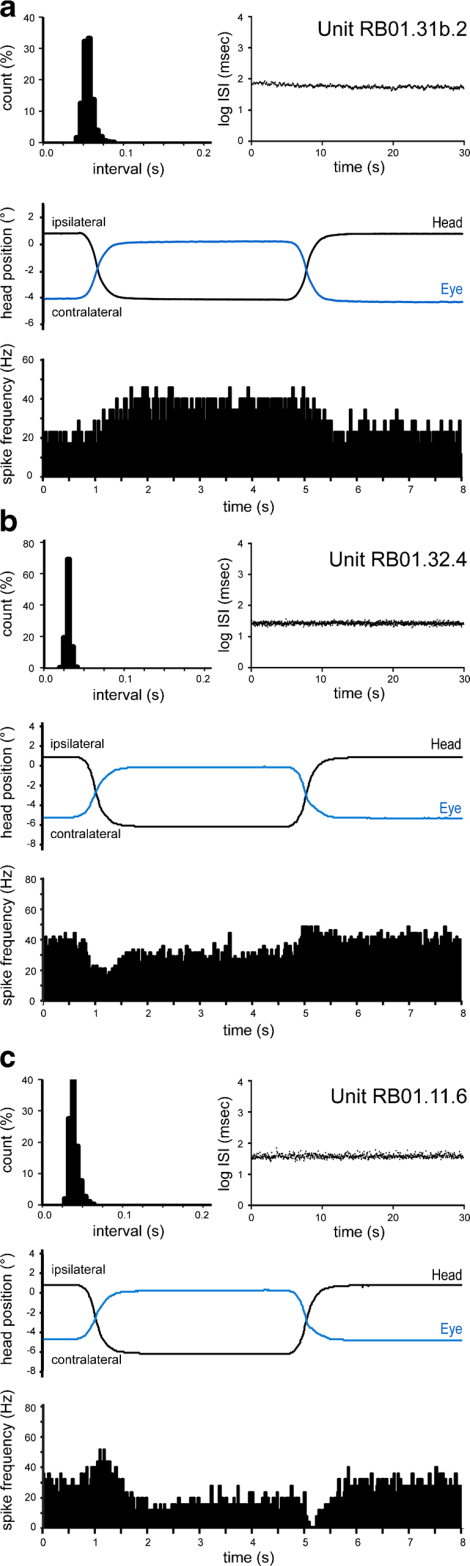
Response properties of floccular UBCs in the awake rabbit. **a** Example (unit RB01.31b.2) that signaled only eye position. *Top left panel*, ISI histogram showing spike intervals during spontaneous activity expressed as normalized counts; *top right panel*, sequential log interspike intervals over a period of 30 s of spontaneous activity; *middle panel*, the sigmoidal head movement provided in the light by a computer-controlled motorized turntable (also used in (**b**) and (**c**)) and the evoked compensatory eye movement (in *blue*); *bottom panel*, modulation profile (average of 7 cycles) in response to the head movement shown in the middle panel. The 0 reference in the time line indicates the time of a trigger pulse in the computer command (also for (**b**) and (**c**)). Panels are arranged similarly in (**b**) and (**c**). **b** Example (unit RB01.32.4) displaying eye position and head velocity profiles with the same response polarity (average of 19 cycles). **c** Example (unit RB01.11.6) that in comparison to the unit shown in Fig. 2b has an oppositely directed velocity profile, but a similarly directed position profile (average of 6 cycles) (Color figure online)

In the flocculus of the awake rabbit, many units had spontaneous activity that adhered to the general features of UBCs recorded in anesthetized animals and were quite different from those of other recorded interneurons including granule cells [12]. Therefore, we are confident that most, if not all, of these units indeed represent UBCs. UBC responses to sigmoidal stimulation, although consistent for any particular unit, were very diverse when regarded as a group. Awake rabbits display compensatory eye movements in response to sigmoidal head movement (Fig. 2). Compensatory eye position changes were an important element of UBC responses in the awake animal. Here, we present three examples of typical response types observed during sigmoidal stimulation in the light.

The first example (unit RB01.31b.2) demonstrated a regular spontaneous firing frequency around 19 Hz (Table 1), which doubled upon moving the turntable in the contralateral direction (Fig. 2a). Note that the response of this unit did not reflect the velocity or the acceleration profile of the movement of either the head or the eye but, rather, reflected the position of the eye. It should be further noted that the response latency (approximately 500 ms) of this unit was considerably longer than that seen in the anesthetized rabbit. Another example of an awake rabbit UBC with similar eye position sensitivity and a quite long latency is shown in our previous publication [13].

The second example (unit RB01.32.4) displayed a different response type (Fig. 2b). This unit demonstrated the characteristics of a vestibular type 1 head-velocity-related response (see Fig. 1a, velocity response, middle trace) and, in addition, showed eye position sensitivity. The firing rate with the eye stationary in a contraversive position was higher than when the eye was in an ipsiversive position (and, in this case, the eye response polarity was opposite to that of the unit shown in Fig. 2a).

Finally, the third example (unit RB01.11.6, Fig. 2c) illustrates a UBC that had a vestibular type 2 head-velocity-related response and also eye position sensitivity. Note that the head velocity response was opposite to that of the unit shown in Fig. 2b, while the polarity of the eye position signal was in the same direction as for the unit in Fig. 2b.

Of the 14 UBCs obtained from awake rabbits, 5 UBCs showed only eye-position-related responses, 8 showed head-velocity-related responses (4 type 1 and 4 type 2), and 1 showed a combination of head-velocity- and head-acceleration-related responses. Four of these nine UBCs also had eye position sensitivity. One type 1 UBC showed a response to eye saccades. For these 14 cells, the average firing rate was 24.0 ± 14.5 Hz (mean ± s.d.). The CV2 was 0.11 ± 0.06 (mean ± s.d.). Compared to anesthetized UBCs, both the average firing rate (*t* = 1.14; *p* = 0.26) and the CV2 (*t* = 0.84; *p* = 0.41) did not significantly differ.

## Discussion

The present study indicates that in both the anesthetized and the awake rabbit, recorded units can be identified as UBCs by comparing their spontaneous firing characteristics to the critical measures determined to represent that cell type [12, 15]. Noting the robustness of these measures, while also taking into account the very different firing characteristics of other units in the flocculus of both anesthetized and awake rabbits (Hensbroek et al., unpublished data), we are confident that these measures indeed identify UBCs. Of course, it seems quite possible that UBCs identified in this way represent only one of the two histochemical UBC subtypes. Indeed, since the type I UBC displays a regular firing pattern in vitro [8], we suggest that our identification procedure may specifically target the calretinin-positive subpopulation. These cells generally are also the somewhat larger UBCs [7, 8], which could at least partly explain why this category may have been over-represented in our sample of juxtacellular recordings [12, 13]. Indeed, our previous studies noted that some morphologically identified UBCs displayed a more irregular pattern that was difficult to characterize [12]. Thus, the present study may not have incorporated units belonging to the type II (mGluR1α) class of UBCs, whose members may nevertheless be abundant in the rabbit flocculus.

Haar et al. [17] have questioned the validity of our decision algorithm. They attempted to evaluate it in two ways: (1) by using a simulated data set based on a sample of statistics with which we developed our algorithm [12] and (2) by applying our algorithm to spontaneous firing statistics from juxtacellularly identified cells collected in the mouse vestibulocerebellum [18]. With respect to the first attempt, Haar et al. [17] created simulated data assuming a multivariate normal distribution of the five statistical parameters used in our decision algorithm [12]. However, the assumption of normality is not supported by our data (see Fig. 8 of [12]). Thus, use of such simulated data is inappropriate. With respect to the second attempted evaluation, juxtacellular data were obtained from compromised cerebella [18] as evidenced by both the abnormally low average Purkinje cell spontaneous complex spike rate (0.2 Hz, far from the universally recognized average value of about 1 Hz) and the abnormally low average spontaneous simple spike rate (20.1 Hz, far from the normal values in mice of about 50 Hz or more; e.g., [19–21]). Furthermore, it is very likely that Haar et al. [17] did not calculate the statistical measures from sufficiently long periods of spontaneous activity. They reported that, of the 92 interneurons made available to them [18], they used only those 45 with spontaneous activity recordings lasting longer than 10 s but that lower limit is substantially shorter than the typically recommended 60 s [12, 15]. For many of these 45 cells, the spontaneous recording times were likely little longer than 10 s and too short for statistical reliability. Finally, there is a serious discrepancy between the number of interneurons said to have been tested in the decision algorithm (*n* = 45) and the number of interneurons (*n* = 87) presented in their Table 1. Where these additional 42 interneurons came from is unexplained, but it raises questions about the inclusion of inappropriate recordings in Table 1. In our view, one can only conclude that Haar et al. [17] did not properly test our decision algorithm. We remain confident of its validity.

The spontaneous-activity-identified UBCs in the anesthetized rabbits showed modulation patterns that reflected either head velocity or different aspects of head acceleration, while in the awake rabbit, UBCs also frequently signaled eye position. The UBC modulation patterns related to acceleration are not expected from the common modulation patterns of mossy fiber inputs from the brainstem. Hence, UBCs may transform velocity-related signals to acceleration-related signals of varying complexity, thus providing granule cells with a wide diversity of kinematic signals.

Response delays of UBCs were long in the anesthetized rabbit (up to 100 ms) and could be far longer, up to 500 ms, in the awake rabbit. These observations may be in line with a recently proposed hypothesis suggesting that UBCs may transform visuo-vestibular input signals to slow motor signals in the control of eye and head movement [11, 18, 22].

In conclusion, the great diversity of UBC responses, in combination with the presumed bias in the type of our identified UBCs, makes it clear that substantial work is still ahead before we understand the role of UBCs in cerebellar operations.
